# Orthostatic hypotension following posterior spinal fusion surgeries for spinal deformity correction in adolescents: prevalence and risk factors

**DOI:** 10.1186/s12891-021-04931-0

**Published:** 2021-12-13

**Authors:** Ying Yang, Yaping Chen, Bingdu Tong, Xue Tian, Chunjie Yu, Zhe Su, Jianguo Zhang

**Affiliations:** Department of Orthopedics of Peking Union Medical College Hospital, 1st Shuai Fu Yuan, Dongcheng District, Beijing, 100730 People’s Republic of China

## Abstract

**Study design:**

Retrospective case series.

**Objectives:**

This study aimed to determine the prevalence and risk factors for orthostatic hypotension (OH) in adolescents undergoing posterior spinal fusion for spinal deformity correction.

**Methods:**

The data of 282 consecutive adolescents who underwent posterior spinal fusion for spinal deformity correction in our center over 12 months were retrieved. Patient characteristics, including whether laminectomy or osteotomy was performed during the surgery, the occurrence of postoperative nausea and vomiting (PONV), perioperative hemoglobin albumin changes, perioperative blood transfusion, length of bed rest, willingness to ambulate, length of postoperative exercises of the lower limbs, and length of hospital stay, were collected and compared statistically between patients who did and did not develop postoperative OH.

**Results:**

Of 282 patients, 197 (69.86%) developed OH postoperatively, and all cases completely resolved 5 days after the first out-of-bed exercises. Significant differences in the incidence of PONV, the willingness to ambulate and the length of postoperative exercises of the lower limbs were observed. The mean length of hospital stay of the patients with OH was longer than that of the patients without OH.

**Conclusion:**

Our study suggests that temporary OH is a common manifestation following posterior spinal fusion for spinal deformity correction in adolescents. Postoperative OH may increase the length of hospital stay in these patients. Patients with PONV, who are not willing to ambulate and who perform postoperative lower limb exercises for a shorter time are more likely to have OH.

## Introduction

Orthostatic hypotension (OH), which is when a patient’s blood pressure falls when moving from a supine position to a standing position, is a common cardiovascular disorder. It is defined as a decrease in the systolic blood pressure (BP) by more than 20 mmHg or a decrease in the diastolic BP be more than 10 mmHg from the baseline BP within 3 min of changing the body position from a supine position to an upright posture [1]. Postoperative OH is a common manifestation following various surgeries, and its etiology is multifactorial and may vary according to the type of surgery [2, 3]. Postoperative OH is often characterized by symptoms of syncope, dizziness, and light headedness. However, weakness, fatigue, cognitive issues, visual blurring, headache, neck pain, orthostatic dyspnea, or chest pain caused by OH may also occur in some patients and make patients distressed, potentially delaying recovery after surgery [4, 5]. An association between OH and many types of surgeries, including cervical spine surgery, has been reported [3, 6, 7]. However, to date, no studies have studied the association between OH and posterior spinal fusion surgeries for the treatment of adolescent spinal deformities. We conducted this study to evaluate the prevalence and risk factors for OH in adolescent patients undergoing posterior spinal fusion for spinal deformity correction.

## Methods

After institutional review board approval was received, all adolescent patients who had undergone posterior spinal fusion surgery under general anesthesia for spinal deformity correction from March 2019 to June 2020 were enrolled. The inclusion criteria were as follows: 1) patients who underwent posterior fusion surgery for spinal deformity correction; 2) patients aged between 10 years and 18 years; 3) patients without dura tears during surgery; 4) patients without a history of OH; and 5) patients without a history of neuromuscular disease or neurological deficits. All patients were asked to perform early ambulation and stand within 72 h after surgery. During the postoperative in-bed period, patients was required to perform lower limb function exercises, including ankle pump exercise, quadriceps muscle strength training, straight-leg-raising movements, and other continuous passive motion exercises. OH is defined as a decrease in the systolic BP by more than 20 mmHg or a decrease in the diastolic BP be more than 10 mmHg from the baseline BP within 3 min of changing the body position from a supine position to an upright posture, and is confirmed by nurses and at least one experienced clinician. The SRS-22 questionnaire was used to evaluate function at 3 months after surgery.

The inpatient records were reviewed for patient characteristics such as age, sex, body mass index (BMI), the number of levels instrumented and fused, whether laminectomy or osteotomy was performed during the surgery, the occurrence of postoperative nausea and vomiting (PONV), perioperative hemoglobin albumin changes, perioperative blood trans fusion, the length of bed rest, the willingness to ambulate, the length of postoperative exercises of the lower limbs, and the length of hospital stay.

Statistical analysis was performed using IBM SPSS Statistics (version 22.0), and significance tests were 2-sided at 5%. The results are expressed as means and SDs for the continuous variables and frequencies for the categorical variables. The *P* values were calculated using independent-samples t tests or nonparametric tests. For the categorical variables, the chi-square or Fisher’s exact test was used. Binary logistic regression analysis was used to identify the risk factors affecting postoperative OH. The significance level was set at α = 0.05.

## Results

**Fig. 1:**
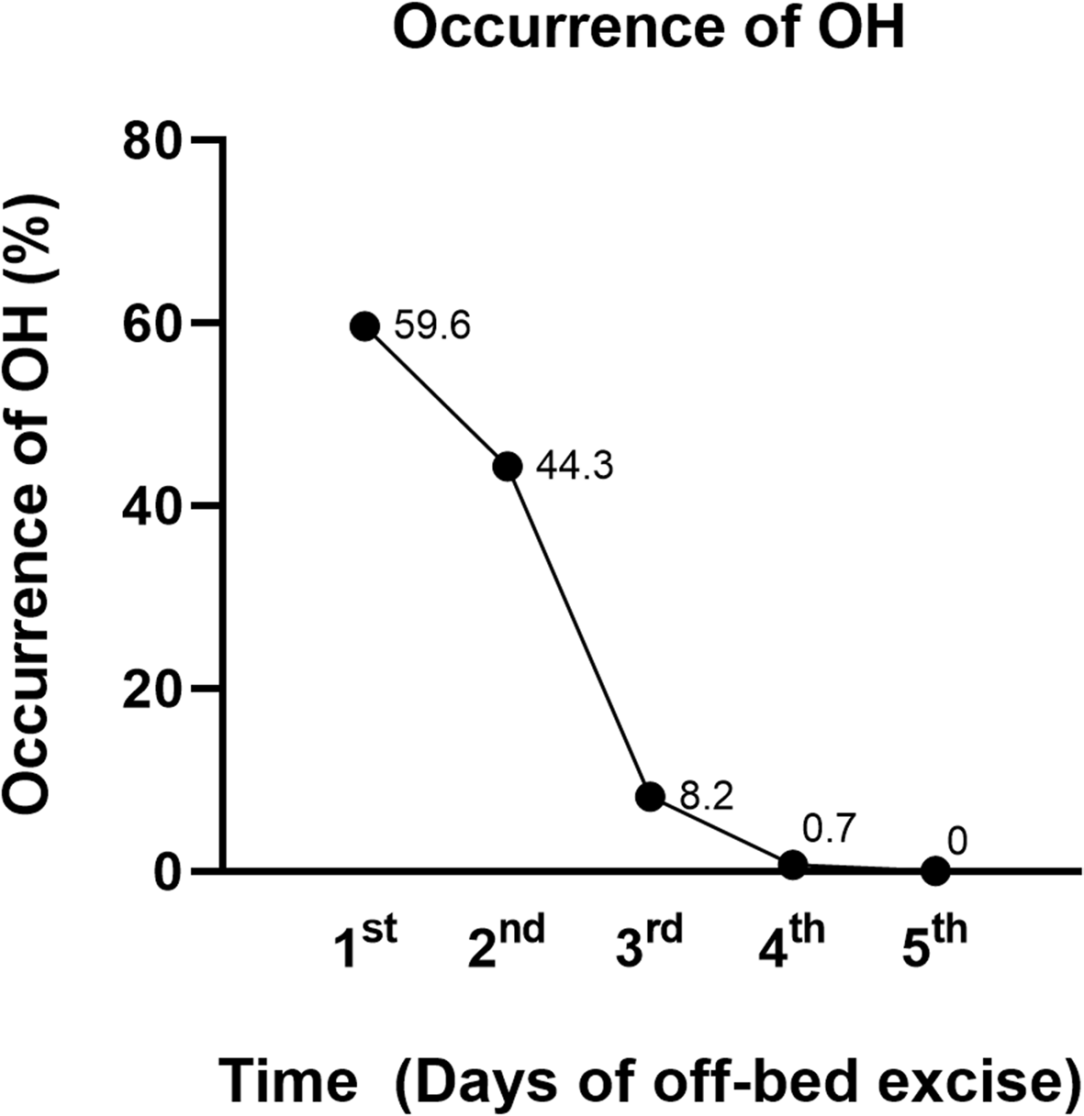
Most postoperative OH cases occurred within 2 days after standing from bed. All OH cases completely resolved after the fifth day of standing

Two hundred and eighty-two patients were enrolled. None of them had perioperative neurological deficits. There were 178 males and 104 females. The mean age was 11.31 ± 3.49 (10–18) years, and postoperative OH occurred in 197 (69.86%) patients. None of the patients developed electrolyte imbalance after surgery. Most postoperative OH cases occurred within 2 days after the patient started standing from bed. All OH cases completely resolved after the fifth day of standing (Fig. 1). One hundred and sixty-eight patients developed OH the first time they stood postoperatively. Among the 114 patients who did not develop OH the first time they stood postoperatively, 29 developed OH when standing from the supine position on a subsequent day (Table 1).

**Table 1:** Timing of the postoperative OH

**OH occurred at the time of 1st postoperative standing**		
**Duration of OH(≤ 2 days)**	154	91.67%
**Duration of OH(> 2 days)**	14	8.33%
**Without OH at the time of 1st postoperative standing**		
**Without OH until discharge**	85	74.56%
**OH occurred during the following Days**	29	25.44%

A t test or chi-square test was performed to compare the variables between patients with and without postoperative OH. All patients received intravenous anesthesia with propofol and fentanyl, and continuously patient controlled analgesia (PCA) with non-steroidal anti-inflammatory drugs (NSAIDs) for postoperative analgesia. Ondansetron hydrochloride and granisetron hydrochloride were used to prevent PONV, while it still occurred in 129 (45.74%) patients. There were no significant differences in age, sex, the number of levels instrumented and fused, whether laminectomy or osteotomy was performed during the surgery, perioperative hemoglobin albumin changes, perioperative blood transfusion or the length of bed rest. Significant differences were found in the occurrence of PONV (*P* = 0.000), willingness to ambulate (*P* = 0.000), length of postoperative exercises of the lower limbs (*P* = 0.000) and BMI (*P* = 0.016). The mean length of hospital stay of the patients with OH was longer than that of the patients without OH (*P* = 0.002). No significant differences were found in the SRS-22 score at 3 months after surgery (*P* = 0.585) (Table 2).

**Table 2:** Comparison between patients with and without postoperative OH

	Cases (n)	With OH	Without OH	Incidence of OH	X^2^/t	*P* value
Gender						
Male	178	120	58	67.42%	1.367	0.282
Female	104	77	27	74.04%		
Occurrence of PONV						
+	129	112	17	86.82%	32.493	0.000
-	153	85	68	55.56%		
Ambulation willingness						
+	180	98	82	54.44%	56.150	0.000
-	102	99	3	97.06%		
Blood transfusion						
+	65	47	18	72.31%	1.830	0.176
–	217	150	67	69.12%		
Length of postoperative Lower-limb exercise						
0-15 min/d	146	142	4	97.26%	136.876	0.000
15-30 min/d	87	49	38	56.32%		
>30 min/d	49	6	43	12.24%		
Osteotomy in surgery						
+	61	48	13	78.69%	2.882	0.090
-	221	149	72	67.42%		
Length of staying in bed(h)		62.37 ± 10.33	61.72 ± 7.55		−0.529	0.597
Age (years)		11.31 ± 3.09	11.31 ± 3.65		0.018	0.986
Height (cm)		1.51 ± 0.19	1.55 ± 0.20		1.631	0.104
Weight (kg)		38.65 ± 14.90	37.48 ± 13.28		−0.784	0.434
Body mass index		18.10 ± 3.75	14.41 ± 3.27		−2.238	0.016
HGB decrease value (g/L)		30.62 ± 11.00	29.10 ± 12.45		−1.021	0.308
ALB decrease value (g/L)		10.17 ± 3.12	9.62 ± 3.24		−1.338	0.182
Surgical duration (min)		228.4 ± 62.28	228.82 ± 54.45		0.054	0.957
Number of fusion segments		8.77 ± 3.90	8.82 ± 3.76		0.104	0.917
Hospital Stay		11.23 ± 3.35	8.10 ± 3.41		28.142	0.002
SRS-22 score		4.3 ± 1.65	4.4 ± 1.29		0.547	0.585

Binary logistic regression was used to evaluate the risk factors for OH that had been identified with the t test or chi-square test. The occurrence of PONV, willingness to ambulate, and time of postoperative exercises of the lower limbs were found to be significantly associated with postoperative OH (Table 3).

**Table 3:** Risk factors of OH (Binary logistic regression)

Factors	Regression coefficients	Standard error	Wald	*P*	Odds ratio	95% Confifidence Interval	
						Lower	Upper
Constant	8.198	1.470	31.086	0.000	–		
PONV	1.786	0.476	14.111	0.000	5.967	2.350	15.154
Ambulation willingness	−2.991	0.694	18.561	0.000	0.050	0.013	0.196
Lower limb exercise time	−2.458	0.354	48.206	0.000	0.086	0.043	0.171
Body mass index	−0.067	0.060	1.238	0.266	0.935	0.831	1.052

## Discussion

OH is a common cardiovascular disorder whose clinical significance is increasingly being recognized, as OH can decrease quality of life and potentially worsen prognoses [8, 9]. OH has been well studied and is closely associated with other common chronic diseases, including hypertension, congestive heart failure, diabetes mellitus, and Parkinson’s disease. The prevalence of OH in patients older than 65 years of age was found to be approximately 20% [10].

Most previous studies on OH were population-based cohort studies or performed in elderly individuals [11, 12]. However, there are few studies on OH in hospitalized patients. Hospitalized patients are particularly vulnerable to the consequences of OH, particularly falls, because postural BP regulation may be disturbed by many common acute illnesses as well as by bed rest and drug treatment [2, 13]. Feldstein reported that OH occurred in as many as 60% of hospitalized adults. Acute physiological and psychological changes due to illness, surgery and anesthesia occur in patients who undergo surgery. Postoperative OH has been well-documented [2]. Hanada et al. found that approximately 40% of 195 patients undergoing cardiothoracic and abdominal surgery experience OH during early postoperative mobilization [7].

Early mobilization is important after major orthopedic surgery to prevent morbidities and long hospital stays. Postoperative OH can prevent early mobilization and has been observed in patients undergoing major orthopedic surgeries. Postoperative OH can lead to failed physiotherapy in patients who have undergone hip arthroplasty, and its incidence has been reported to be 40–50% [6, 14]. Associations between OH and spine surgeries and spinal cord injuries have also been found. Edward et al. found that 22 of 190 patients who underwent cervical spine surgery (11.6%) developed postoperative OH. The authors found that the presence of neurological deficits is a risk factor for postoperative OH [3]. The link between spinal cord injuries (SCIs) and the development of OH has been observed [15]. Postural changes during physiotherapy and mobilization have been shown to induce clinically significant OH in 74% of SCI patients, with symptoms in 59% of patients [15]. Significant differences in the incidence of OH between patients with compressive cervical myelopathy and healthy controls were found in Srihari’s study [16]. Furthermore, McKinley et al. found that traumatic SCI patients had a significantly higher incidence of OH than did nontraumatic SCI patients (36.7% vs 5.3%) [17].

Previous studies have shown that early ambulation can decrease the length of hospital stay and perioperative complications and improve functional outcomes in adolescent and adult patients undergoing correction surgery for spinal deformities [18, 19]. Thus, we asked patients in this group to perform out-of-bed exercises and stand within 72 h after surgery. Postoperative OH may occur and affect recovery in these patients. However, until now, there have been no studies on postoperative OH following posterior spinal fusion surgeries for spinal deformity correction in adolescents. In the current study, we determined the incidence of postoperative OH following posterior spinal fusion for the treatment of adolescent spinal deformities. One hundred and nighty-seven (69.86%) of 282 adolescent patients who underwent correction surgeries for spinal deformities and performed out-of-bed exercises early and stood within 72 h after surgery developed postoperative OH, which mostly occurred within 2 days of standing, and all cases completely resolved after the fifth day of standing. The presence of postoperative OH significantly increased the length of hospital stay. However, no differences in the SRS-22 score were found between the patients with and without postoperative OH.

The risk factors for postoperative OH following spine surgeries remain unclear. Neurological deficits and traumatic SCI have been reported to be associated with a higher incidence of postoperative OH [3]. In our study, we found that age, sex, the number of levels instrumented and fused, whether laminectomy or osteotomy was performed during the surgery, the postoperative hemoglobin level and the postoperative albumin level were not significantly associated with postoperative OH following posterior spinal fusion surgeries for the correction of spinal deformities in adolescent patients. Significant associations between PONV, willingness to ambulate, length of postoperative exercises of the lower limbs and postoperative OH were found. The association between PONV and OH was reported in a previous study. Franz et al. found that female patients with preoperative OH had an increased risk of PONV [20]. For patients with risk factors for PONV, including the female sex, a history of motion sickness or previous PONV, a nonsmoking status, and the use of postoperative opioid drugs, therapies should be given to decrease the occurrence or severity of PONV and postoperative OH. Early postoperative exercise, including lower limb strength training, is an essential component of rehabilitation protocols following posterior spinal fusion surgeries and can improve function and shorten the hospital stay [21–23]. Although a statistical difference was found in BMI between two groups (*P* = 0.016), there was no significance between BMI and the occurrence of postoperative OH in further binary logistic regression (*P* = 0.266). Therefore, it is inappropriate to include BMI as a risk factor for OH. In previous studies, no relationship between occurrence of OH after spinal surgery and BMI has been reported. In Parkinson’s disease, low BMI is considered to be a risk factor for OH [24, 25], which is consistent with a study on risk factors for OH in elder people [26]. This is considered with reduced autonomic function and lower leptin levels (a hormone produced by fat cells that possibly mediating the inhibition of sympathetic excitation). In a study of young adults with posttraumatic stress disorder (PTSD), higher BMI was statistically associated with OH (30.13 ± 6.29 vs 28.07 ± 6.53, *P* = 0.02) [27]. However, considering the involvements of smoking, alcohol addiction, and using of psychotropics, this association is thought to be tenuous. Since all participants in our study were adolescents and patients with neuromuscular disease or neurological deficits were excluded, more exploration between postoperative OH and BMI is needed. According to our findings, better patient education of early postoperative ambulation to encourage patients to perform early postoperative out-of-bed exercises and postoperative strategies to increase the mobility of the lower limbs may be helpful to decrease the incidence of postoperative OH following posterior spinal fusion surgeries for the treatment of adolescent spinal deformities.

## Conclusion

Temporary OH is a common manifestation following posterior spinal fusion for spinal deformity correction in adolescents, and all patients’ OH completely resolved after the fifth day of out-of-bed activity. Postoperative OH may increase the length of hospital stay in these patients. Patients with PONV, who are not willing to perform out-of-bed exercises and perform postoperative lower limb exercises for a shorter time are more likely to have postoperative OH. Hence, we suggest that postural BP should be routinely monitored in this group of patients so that early intervention can be initiated. Strategies to prevent PONV, to improve patients’ willingness to ambulate and to increase the length of postoperative lower limb exercises may be helpful to decrease the occurrence of postoperative OH in these patients.

## Data Availability

The datasets generated and analyzed during the current study are not publicly available due risk of compromising individual privacy but are available from the corresponding author on reasonable request.
